# Evaluation of superparamagnetic iron oxide-polymer composite microcapsules for magnetic resonance-guided high-intensity focused ultrasound cancer surgery

**DOI:** 10.1186/1471-2407-14-800

**Published:** 2014-11-03

**Authors:** Yang Sun, Yuanyi Zheng, Pan Li, Dong Wang, Chengcheng Niu, Yuping Gong, Rongzhong Huang, Zhibiao Wang, Zhigang Wang, Haitao Ran

**Affiliations:** Second Affiliated Hospital, Institute of Ultrasound Imaging, Chongqing Medical University, Chongqing, P. R. China; College of Biomedical Engineering, Chongqing Medical University, Chongqing, P. R. China

**Keywords:** Cancer surgery, Hyperthermia, HIFU, MRI guidance, Iron oxide, Polymer

## Abstract

**Background:**

Superparamagnetic poly (lactic-co-glycolic acid) (PLGA)-coated Fe_3_O_4_ microcapsules are receiving increased attention as potential diagnostic and therapeutic modalities in the field of oncology. In this study, PLGA-coated Fe_3_O_4_ microcapsules were combined with a magnetic resonance imaging-guided high-intensity focused ultrasound (MR-guided HIFU) platform, with the objective of investigating the effects of these composite microcapsules regarding MR-guided HIFU liver cancer surgery *in vivo*.

**Methods:**

PLGA-coated Fe_3_O_4_ microcapsules consisting of a liquid core and a PLGA-Fe_3_O_4_ shell were fabricated using a modified double emulsion evaporation method. Their acute biosafety was confirmed *in vitro* using MDA cells and *in vivo* using rabbits. To perform MR-guided HIFU surgery, the microcapsules were intravenously injected into a rabbit liver tumor model before MR-guided HIFU. T_2_-weighted images and MR signal intensity in normal liver parenchyma and tumor tissue were acquired before and after injection, to assess the MR imaging ability of the microcapsules. After MR-guided HIFU ablation tissue temperature mapping, the coagulative volume and histopathology of the tumor tissue were analyzed to investigate the ablation effects of MR-guided HIFUs.

**Results:**

Scanning and transmission electron microscopy showed that the microcapsules displayed a spherical morphology and a shell-core structure (mean diameter, 587 nm). The hysteresis curve displayed the typical superparamagnetic properties of the microcapsules, which are critical to their application in MR-guided HIFU surgery. In MR-guided HIFU surgery, these microcapsules functioned as an MRI contrast agent, induced significant hyperthermal enhancement (*P* < 0.05) and significantly enhanced the volume of coagulative necrosis (*P* < 0.05).

**Conclusions:**

The administration of PLGA-coated Fe_3_O_4_ microcapsules is a potentially synergistic technique regarding the enhancement of MR-guided HIFU cancer surgery.

**Electronic supplementary material:**

The online version of this article (doi:10.1186/1471-2407-14-800) contains supplementary material, which is available to authorized users.

## Background

The clinical application of ultrasound is no longer limited to diagnosis. High-intensity focused ultrasound (HIFU) is a newly developed technique that applies ultrasonic energy to a focused region for the hyperthermal treatment of solid tumors [1, 2]. Compared with conventional surgical, chemotherapeutic and radiotherapeutic approaches, HIFU is a logical and attractive treatment modality that can selectively and non-invasively destroy multiple foci of origin [3, 4]. Moreover, HIFU provides additional therapeutic options in cases where conventional therapies have failed [5–7].

HIFU performance critically relies on imaging quality for an accurate determination of tumor location to facilitate the optimal deposition of ultrasonic energy in the tumor. The integration of magnetic resonance imaging (MRI) and HIFU forms an MR-guided HIFU system that offers superior soft tissue MRI resolution combined with non-invasive real-time tissue temperature mapping (T-Map) capabilities [8–10]. Consequently, MR-guided HIFU is being increasingly used in the clinic [11–14]. Despite its growing clinical acceptance, MR-guided HIFU has two key limitations. First, MRI as used in MR-guided HIFU can fail to visualize small early stage or MRI-insensitive tumors [15–17]. Second, the ultrasonic energy emitted by the MR-guided HIFU transducer is considerably attenuated when shifting from an *in vitro* environment to *in vivo* tissue; this attenuation adversely affects MR-guided HIFU ablation efficiency because the ability of HIFU to successfully ablate tumors depends on its capacity to deposit energy in tissue [18–21]. Therefore, limitations in tumor visualization and *in vivo* energy deposition adversely affect the treatment efficacy of MR-guided HIFU.

Superparamagnetic iron-oxide nanoparticles (SPIONs) possess unique magnetic properties that make them attractive advanced biomaterial candidates [22–25]. In cancer diagnosis and therapy, SPIONs can serve as MRI contrast agents [26, 27], miniaturized heaters capable of destroying malignant cells and colloidal carriers for targeted drug delivery [28–30]. Since SPION-enhanced MR imaging can be used to monitor the tumor prior to ablation therapy, SPIONs are particularly suitable for MR-guided HIFU applications. Moreover, as a functional medium, SPIONs can also change the acoustic tissue microenvironment in the targeted region, thereby enhancing the tumor-ablative effects of MR-guided HIFUs.

The objective of the present study was to combine the merits of SPIONs and polymers by constructing a composite particle, namely the superparamagnetic poly (lactic-co-glycolic acid) (PLGA)-coated Fe_3_O_4_ microcapsule. We investigated the *in vitro* properties of these superparamagnetic PLGA-coated Fe_3_O_4_ microcapsules and the *in vivo* application of these microcapsules in MR-guided HIFU liver cancer surgery using a rabbit model.

## Methods

### Synthesis of PLGA-coated Fe_3_O_4_ microcapsules

Preparation and storage of the microcapsules were performed in the dark. Briefly, a 200-ml solution (3.1% w/v) of nano-sized Fe_3_O_4_ particles (31 mg/ml; size, 10 nm; Ocean NanoTech, USA) was added to 2 ml of CH_3_Cl dissolved in 100 mg of PLGA (50:50; MW = 20000; Daigang, China). For cell incubation, the fluorescent dye DiI was incorporated into the composite microcapsules. The above mixture was emulsified (Sonics & Materials Inc., USA) for 45 s. After adding 200 ml of deionized water, the solution was homogenized (FJ300-SH, Shanghai, China) for 5 min with a 10-ml poly(vinyl alcohol) (PVA; MW = 25000; Sigma) solution (5% w/v). Then, the CH_3_Cl solution was removed by mechanical mixing for 2 h. The mixture was subsequently centrifuged at 800 rpm for 10 min. After centrifugation, the precipitate containing large microcapsules was discarded, and the functionalized PLGA-coated Fe_3_O_4_ microcapsules were generated by a second centrifugation of the remaining microcapsule suspension at 5000 rpm for 5 min. In addition, pure PLGA microcapsules were prepared without the addition of Fe_3_O_4_ particles and used as a control agent.

### Characterizing PLGA-coated Fe_3_O_4_ microcapsules

The average size of the PLGA-coated Fe_3_O_4_ microcapsules was characterized using the Laser Particle Size Analyzer System (Zeta SIZER 3000HS: Malvern, PA, USA). The morphological and structural characteristics of the microcapsules were estimated using scanning electron microscopy (SEM) (S-3400 N: Hitachi, Japan) and transmission electron microscopy (TEM) (H-7500: Hitachi, Japan). DiI-labeled PLGA microcapsules were observed using inverted fluorescence microscopy (Olympus IX71: Canada). The magnetic properties of the microcapsules were investigated using the Physical Property Measurement System (PPMS, Model 6000: Quantum Design).

### MDA cell culture and PLGA-coated Fe_3_O_4_ microcapsule uptake by MDA cells

MDA cells obtained from the American Type Culture Collection (ATCC, USA) were cultured in RPMI-1640 medium supplemented with 10% FBS (both from Hycline) at 37°C with 5% CO_2_ in a humidified atmosphere and passaged every 2–3 days. DiI-labeled PLGA-coated Fe_3_O_4_ microcapsules were irradiated using a Co^60^ gamma ray source for sterilization prior to incubation with MDA cells.

MDA cells (2 × 10^5^ per well) were placed on six-well tissue-culture clusters 24 h before incubation with PLGA-coated Fe_3_O_4_ microcapsules. Immediately before initiating incubation, the medium was removed from each well and the cells were washed three times with PBS. They were then incubated with the DiI-labeled PLGA-coated Fe_3_O_4_ microcapsules as described below. The microcapsules were diluted to a final Fe_3_O_4_ concentration of 0.15 mg/ml with culture medium and subsequently added to each well. Cells in culture medium without agents were used as the control group. The cells with PLGA-coated Fe_3_O_4_ were individually cultured for 4, 12 and 24 h, while the control group was cultured for 24 h. Thereafter, the medium was removed from each well, and the cells were washed three times with PBS and fixed in 10% formalin solution for 10 min. The cells were then stained using FITC for 45 min and Hoechst for 30 min before observation under an inverted fluorescence microscope.

### MTT assay

Cell viability was determined using the MTT test. MDA cells (1 × 10^4^ per well) were seeded into 96-well plates. After incubation for 24 h, the medium was removed and replaced with fresh culture medium containing PLGA-coated Fe_3_O_4_ microcapsules at Fe_3_O_4_ concentrations of 0.5, 1.0, 2.0, 4.0 and 8.0 mg/ml. Cells in culture medium without agents were used as the control group. Following 24 h incubation, cell viability was measured by the addition of 20 μl 3-(4,5-dimethylthiazol-2-yl)-2, 5-diphenyltetrazolium bromide (MTT; 5 mg/ml) solution for 4 h. Then, 150 μl of DMSO was added to dissolve the formazan crystals. To assess cell viability, optical density (OD) was measured at 490 nm with an enzyme-linked immunosorbent assay (ELISA) plate reader.

### Acute biosafety of PLGA-coated Fe_3_O_4_ microcapsules

Thirty-six New Zealand white rabbits (weight, 2.0–2.5 kg; age, 2–3 months) were purchased and maintained in the Animal Center of Chongqing Medical University under standard conditions in accordance with the university’s environmental guidelines. All animal experiments were approved by the Animal Ethics Committee of Chongqing Medical University. All animal experiments and procedures were performed under complete anesthesia.

Prior to tumor implantation, 18 rabbits were divided into three groups to determine a safe dosage of PLGA-coated Fe_3_O_4_ microcapsules. Each group received a 2-ml injection of a different concentration of PLGA-coated Fe_3_O_4_ microcapsules via the ear vein, at Fe_3_O_4_ concentrations of 1, 4 and 8 mg/ml. Serum was sampled from the rabbits to detect biochemical indicators of liver, kidney and cardiac function before injection and at 1, 3 and 7 days after injection of the PLGA-coated Fe_3_O_4_ microcapsules. These biochemical indicators included alanine aminotransferase (ALT), aspartate aminotransferase (AST), total bilirubin (TBIL), blood urea nitrogen (BUN), creatinine (SCr), creatine kinase (CK) and lactate dehydrogenase (LDH).

### Animal model and experimental equipment

Rabbits with a VX2 tumor located in the thigh were obtained from the Ultrasound Engineering Institute of Chongqing Medical University (Chongqing Medical University). The liver tumor model was developed according to a previously described method [31].

The 18 recipient rabbits with detectable liver cancer (21 days after VX2 tumor implantation) underwent MR-guided HIFU treatment using Symphony A Tim 1.5 T MR-guided HIFU tumor ablation equipment (therapeutic transducer focal length, 145 mm; diameter, 220 mm; operating frequency, 0.94 MHz; Chongqing Haifu Technology, Chongqing, China). This system uses a focused ultrasonic transducer that emits high-intensity ultrasonic energy to target and destroy the tissue-of-interest, while the diagnostic MR scanner images the tumor and monitors the targeted tissue temperature during the therapeutic process.

### MR-guided HIFU surgery for rabbits bearing the VX2 liver tumor

The 18 recipient rabbits with VX2 liver tumors were placed on the MR-guided HIFU treatment bed in the prone position after being completely anesthetized. Their abdomens were entirely immersed in degassed water. The rabbits were randomly divided into three groups: (i) MR-guided HIFU treatment without microcapsules (group I; n = 6); (ii) MR-guided HIFU treatment with a 2-ml intravenous injection of pure PLGA microcapsules at a PLGA concentration of 50 mg/ml (group II; n = 6); and (iii) MR-guided HIFU treatment with a 2-ml intravenous injection of PLGA-coated Fe_3_O_4_ microcapsules at a Fe_3_O_4_ concentration of 3.1 mg/ml (group III; n = 6). Prior to MR-guided HIFU ablation, T_2_-weighted images were acquired before and at 5 min after ear vein injection of pure PLGA microcapsules (group II) and PLGA-coated Fe_3_O_4_ microcapsules (group III). No injection of microcapsules was administered to rabbits in group I; thus, in group I, the second image was acquired at 5 min after the acquisition of the first image. Additionally, the MR signal intensity (SI) within the region of interest (including both normal liver parenchyma and liver tumor) was measured before and at 2 and 5 min after injection of the various agents to assess the enhanced MR imaging ability. After MR scanning, MR-guided HIFU ablation was performed using the “ablated-dot” mode, in which each tumor was destroyed in a single exposure. In all three MR-guided HIFU groups (groups I, II and III), MR-guided HIFU ablation parameters were kept constant with a 250-W acoustic power and a 5-s exposure duration. During MR-guided HIFU ablation, T-Map was imaged in the targeted region to investigate the effects of MR-guided HIFU ablation. To acquire T_2_-weighted images, turbo spin echo (TSE) sequences were run at TR values of 4100 ms (TE, 113 ms; FOV, 300 mm × 300 mm; slice thickness, 4.0 mm).

Animals were sacrificed after MR-guided HIFU ablation, and the livers were immediately removed for macroscopic observation. The tumor along with the surrounding normal liver tissue was sectioned into 2-mm slices, and the maximal section of necrotic tumor tissue was selected for observation of the area of coagulative necrosis. Then, the length, width and depth of the necrotic tissue were compiled from each tissue slice to calculate the volume of coagulative necrosis. The volume of coagulated tissues in the liver tumor was calculated using the following equation: V = π/6 × L × W × D (L, length; W, width; D, depth) [32]. In addition, TEM was performed on the ablated tissue from each tumor to detect ultrastructural changes in the cancerous tissue.

### Statistical analysis

All data are expressed as the mean ± standard deviation. Various groups were compared for differences using one-way analysis of variance (one-way ANOVA). All analyses were performed using SPSS Statistics 19.0. A difference with a *P*-value of <0.05 was deemed statistically significant.

## Results and discussion

### Characterization of PLGA-coated Fe_3_O_4_ microcapsules

According to the microcapsule size distribution (Additional file 1), the mean diameter of the prepared PLGA-coated Fe_3_O_4_ microcapsules was 587 ± 60 nm. The characteristic pore cutoff size ranged from 380 to 780 nm and has been demonstrated in a variety of tumors, although some tumors show pore sizes of ≤2 μm [33–35]. In our study, the smallest particles (approximately 10–20% of the total particles) in the distribution could enter the tumor tissue by means of the enhanced permeability and retention (EPR) effect, which allows extravasation of nanoparticles through large inter-endothelial gaps in the effective tumor microvasculature for the induction of the MR signal and HIFU synergistic therapy.

**Figure 1:**
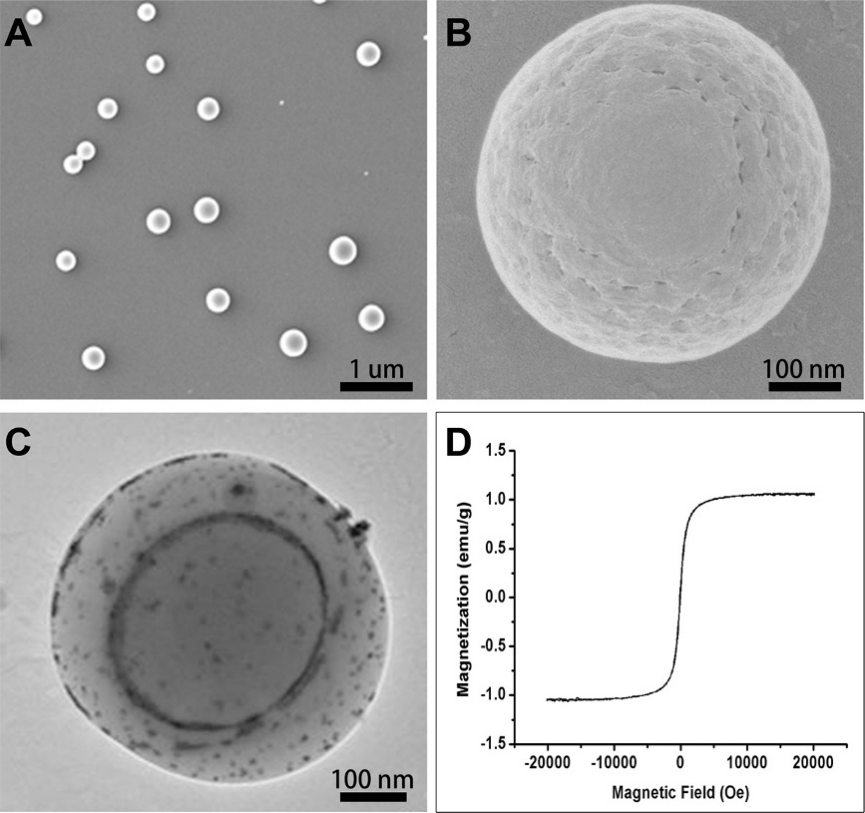
**The characterization of superparamagnetic PLGA-coated Fe**_**3**_ **O**_**4**_ **microcapsules. (A, B)** Scanning electron microscope images of the PLGA-coated Fe_3_O_4_ microcapsules at different magnifications. **(C)** Transmission electron microscope images of the PLGA-coated Fe_3_O_4_ microcapsules. **(D)** Magnetic properties of the superparamagnetic PLGA-coated Fe_3_O_4_ microcapsules (M–H magnetization curve).

Under SEM imaging, the prepared microcapsules displayed a spherical morphology and a non-smooth surface (Figure 1A and B). Because of their organic solubility and small size, the Fe_3_O_4_ nanoparticles embedded themselves in the PLGA shell structure when introduced into the reaction medium prior to the addition of the PVA molecules in the second emulsion. This structure was confirmed by TEM imaging (Figure 1C), where the Fe_3_O_4_ nanoparticles were observed on the surface of the microcapsules as a result of their high electron intensity. The hysteresis curve (Figure 1D) demonstrated the typical superparamagnetic properties of the PLGA-coated Fe_3_O_4_ microcapsules, which are critical to their application in MR-guided HIFU surgery.

**Figure 2:**
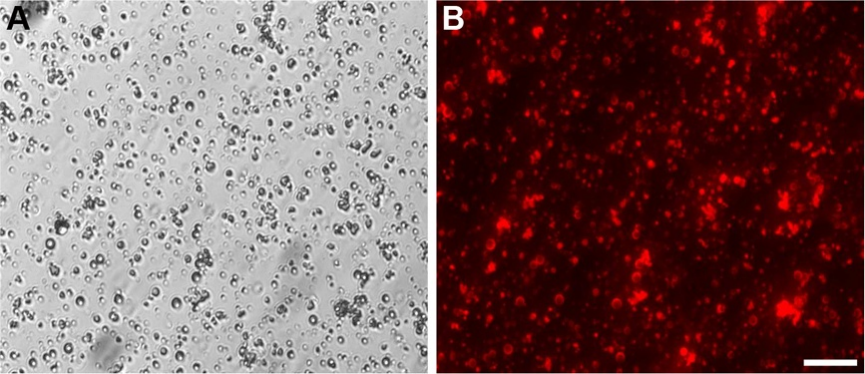
**Morphology of DiI-labeled PLGA-coated Fe**_**3**_ **O**_**4**_ **microcapsules. (A)** Appearance of microcapsules under light microscopy. **(B)** Appearance of microcapsules under fluorescence microscopy. Scale bar: 10 μm.

DiI is a non-cytotoxic lipophilic orange-red fluorescent dye that does not affect cell growth and proliferation. In the preparation process, DiI was miscible with PLGA and Fe_3_O_4_ in chloroform during the formation of the shell of the composite microcapsules. Using fluorescence microscopy, the surface of the microcapsules displayed the ring-shaped orange-red fluorescent dye DiI (Figure 2).

### Uptake of PLGA-coated Fe_3_O_4_ microcapsules by MDA cells

**Figure 3:**
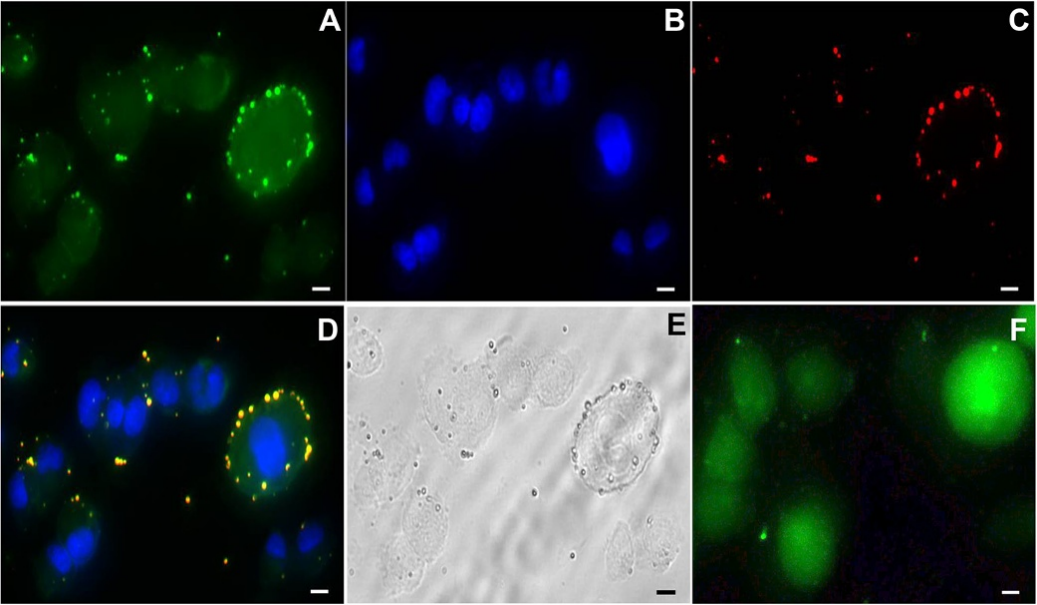
**Uptake of PLGA-coated Fe**_**3**_ **O**_**4**_ **microcapsules by MDA cells observed under fluorescence microscopy. (A)** MDA cells after incubation with DiI-labeled PLGA-coated Fe_3_O_4_ microcapsules for 4 h followed by FITC staining for 45 min. The cytoplasm appears green. **(B)** MDA cells after staining with Hoechst for 30 min with the nucleus appearing blue. **(C)** DiI-labeled PLGA-coated Fe_3_O_4_ with a red appearance under fluorescence microscopy. **(D)** Merged images of **(A)**, **(B)** and **(C)**. **(E)** Appearance of microcapsules under light microscopy. **(F)** MDA cells after staining with FITC in the absence of PLGA particles. Scale bar: 10 μm.

To observe the cellular uptake of PLGA-coated Fe_3_O_4_ microcapsules, as well as the impact of these microcapsules on cell viability, MDA cells were incubated with DiI-labeled PLGA-coated Fe_3_O_4_ over different periods of time. Fluorescence microscopy demonstrated that PLGA-coated Fe_3_O_4_ microcapsules were phagocytosed by MDA cells (Figure 3), with the number of phagocytosed microcapsules increasing with incubation time (12 and 24 h). In Figure 3, MDA cells were stained with FITC and Hoechst for 45 min and 30 min, respectively, after incubation with DiI-labeled PLGA-coated Fe_3_O_4_ microcapsules for 4 h. After staining, the cytoplasm of the MDA cells appeared green (Figure 3A), the nucleus appeared blue (Figure 3B), and DiI-labeled PLGA-coated Fe_3_O_4_ appeared red (Figure 3C) under fluorescence microscopy. There appear to be green dots in Figure 3A in the same place as the dots in Figure 3C. We suspect that these dots were PLGA particles that were stained by FITC; in addition, we could see the merged color in the same location (Figure 3D) but we could not observe these green dots in MDA cells without incubation of the PLGA particles after staining with FITC (Figure 3F). Thus, we believe that FITC has the ability to label PLGA particles.

**Figure 4:**
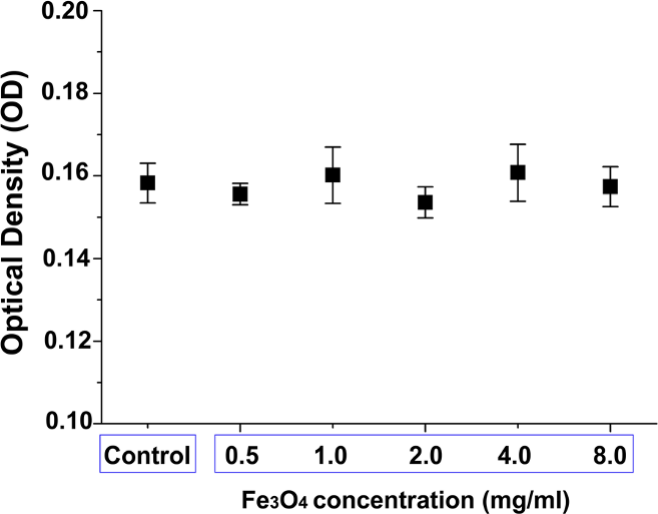
**Cytotoxic effects of PLGA-coated Fe**_**3**_ **O**_**4**_ **microcapsules on MDA cells.** Optical density (OD) was measured at 490 nm in MDA cells incubated with different concentrations of PLGA-coated Fe_3_O_4_ to assess cell viability. The higher the OD, the greater the cell viability. The OD of MDA cells incubated with PLGA-coated Fe_3_O_4_ microcapsules at concentrations of 0.5, 1.0, 2.0, 4.0 and 8.0 mg/ml were 0.156 ± 0.003, 0.160 ± 0.007, 0.154 ± 0.004 and 0.161 ± 0.007, 0.157 ± 0.005, respectively; the OD of the control group was 0.158 ± 0.005. Results showed no significant differences between the PLGA-coated Fe_3_O_4_ groups and the control group (*P* > 0.05; MDA cells in culture medium without agents were used as the control group.

In addition, the OD of MDA cells incubated with PLGA-coated Fe_3_O_4_ at concentrations of 0.5, 1.0, 2.0, 4.0 and 8.0 mg/ml were 0.156 ± 0.003, 0.160 ± 0.007, 0.154 ± 0.004, 0.161 ± 0.007 and 0.157 ± 0.005, respectively; the OD of the control group was 0.158 ± 0.005. The MTT assay indicated that the phagocytosed microcapsules had no significant effect on cell viability relative to the controls (*P* > 0.05; Figure 4).

### Acute biosafety of PLGA-coated Fe_3_O_4_ microcapsules

We injected the PLGA-coated Fe3O4 microcapsules into rabbits to investigate their acute biosafety *in vivo*. After injection of three different concentrations of the microcapsules (Fe_3_O_4_ concentrations of 1 mg/ml, 4 mg/ml and 8 mg/ml), there were no obvious abnormalities in all three groups of rabbits. The serum biochemical indicators (Tables 1, 2 and 3) demonstrated that liver, kidney and cardiac function in the three groups showed no significant changes (*P* > 0.05) at the different post-injection time points (1, 3 and 7 days).

**Table 1:** **Serum biochemical indicators after the injection of PLGA-coated Fe**_**3**_ **O**_**4**_ **microcapsules (1 mg/ml)**

	Pre-injection	1 d	3 d	7 d
ALT (U/L)	64.33 ± 18.34	56.67 ± 15.01	51.00 ± 13.89	54.00 ± 16.09
AST (U/L)	41.33 ± 1.53	42.33 ± 2.52	40.67 ± 4.16	45.33 ± 2.08
TBIL (μmol/L)	3.47 ± 0.40	3.53 ± 0.15	3.27 ± 0.12	3.20 ± 0.26
BUN (mmol/L)	7.55 ± 0.38	8.42 ± 0.39	7.81 ± 0.77	7.50 ± 0.62
SCr (μmol/L)	68.77 ± 7.60	73.70 ± 4.40	62.37 ± 4.10	75.30 ± 9.04
CK (U/L)	3242.67 ± 71.14	3420.00 ± 172.50	3457.67 ± 132.50	3362.33 ± 116.11
LDH (U/L)	839.67 ± 10.02	850.67 ± 19.86	834.00 ± 18.00	857.00 ± 12.77

Note: The same indicators at different time points for pairwise comparisons (*P* > 0.05 for all of the comparisons).

**Table 2:** **Serum biochemical indicators after the injection of PLGA-coated Fe**_**3**_ **O**_**4**_ **microcapsules (4 mg/ml)**

	Pre-injection	1 d	3 d	7 d
ALT (U/L)	50.80 ± 14.23	57.17 ± 14.06	63.53 ± 17.35	53.70 ± 15.11
AST (U/L)	43.33 ± 1.56	41.35 ± 2.46	41.63 ± 3.56	43.83 ± 2.15
TBIL (μmol/L)	3.45 ± 0.39	3.54 ± 0.17	3.29 ± 0.14	3.21 ± 0.33
BUN (mmol/L)	7.79 ± 0.67	8.45 ± 0.37	7.65 ± 0.48	7.66 ± 0.58
SCr (μmol/L)	66.57 ± 6.70	72.73 ± 4.37	64.26 ± 4.11	74.80 ± 8.05
CK (U/L)	3354.39 ± 96.61	3422.00 ± 112.53	3446.62 ± 122.30	3277.65 ± 69.40
LDH (U/L)	841.67 ± 11.02	848.63 ± 17.83	823.14 ± 16.73	856.37 ± 13.42

Note: The same indicators at different time points for pairwise comparisons (*P* > 0.05 for all of the comparisons).

**Table 3:** **Serum biochemical indicators after the injection of PLGA-coated Fe**_**3**_ **O**_**4**_ **microcapsules (8 mg/ml)**

	Pre-injection	1 d	3 d	7 d
ALT (U/L)	59.23 ± 13.77	56.15 ± 14.55	51.30 ± 13.87	61.23 ± 16.55
AST (U/L)	42.88 ± 1.45	41.09 ± 1.65	41.64 ± 2.00	43.56 ± 3.15
TBIL (μmol/L)	3.49 ± 0.38	3.48 ± 0.23	3.37 ± 0.34	3.23 ± 0.32
BUN (mmol/L)	7.58 ± 0.32	8.44 ± 0.43	7.76 ± 0.53	7.59 ± 0.63
SCr (μmol/L)	69.65 ± 5.74	71.88 ± 4.43	66.30 ± 5.31	75.79 ± 7.98
CK (U/L)	3280.98 ± 66.33	3445.09 ± 98.71	3453.98 ± 112.27	3325.0 ± 111.20
LDH (U/L)	853.34 ± 10.71	847.72 ± 15.30	838.99 ± 17.90	850.08 ± 16.98

Note: The same indicators at different time points for pairwise comparisons (*P* > 0.05 for all of the comparisons).

### PLGA-coated Fe_3_O_4_ microcapsules as contrast agents for MR-guided HIFU surgery

**Figure 5:**
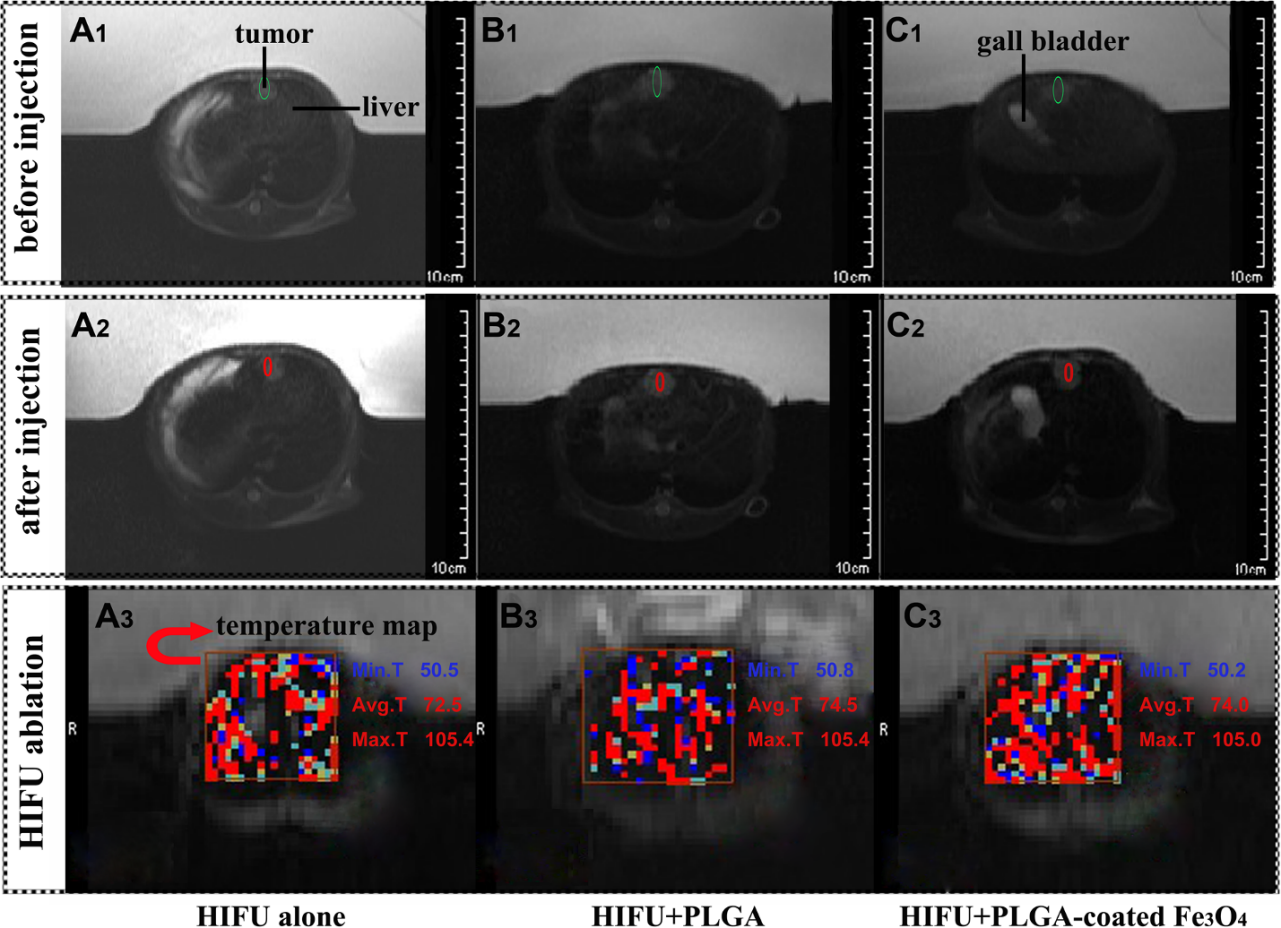
**MR-guided HIFU liver cancer surgery (six rabbits were used in each group). (A)** MR-guided HIFU alone. **(B)** MR-guided HIFU + pure PLGA microcapsules. **(C)** MR-guided HIFU + PLGA-coated Fe_3_O_4_ microcapsules. **(A**_**1**_ **/ B**_**1**_ **/C**_**1**_ **)** T_2_-weighted images of the targeted tissue before MR-guided HIFU ablation (the green circle indicates the tumor site). **(A**_**2**_ **/ B**_**2**_ **/ C**_**2**_ **)** T_2_-weighted images of the targeted tissue at 5 min after the injection of different agents (the red circle identifies the region that HIFU was focused upon). **(A**_**3**_ **, B**_**3**_ **, C**_**3**_ **)** Tissue temperature mapping during MR-guided HIFU ablation. minT, minimum temperature; avgT, average temperature; maxT, maximum temperature.

**Figure 6:**
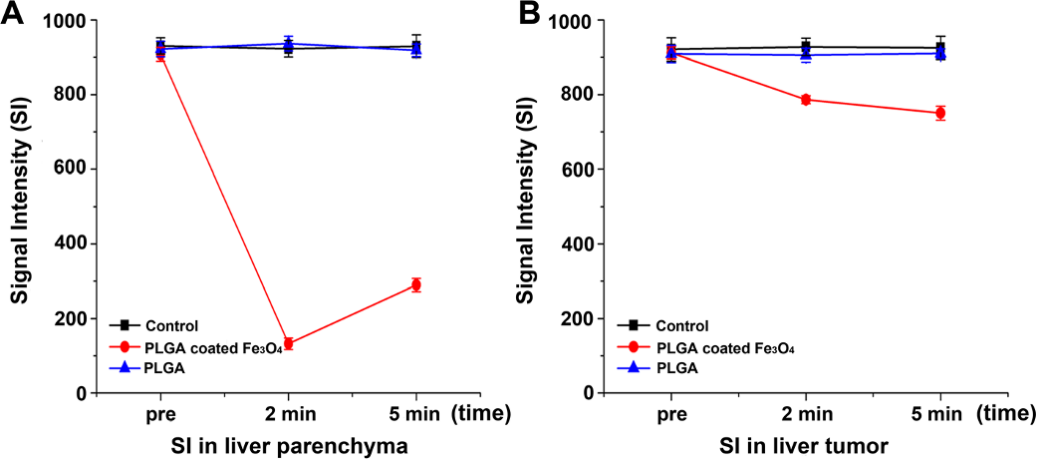
**Analysis of MR signal intensity.** The MR signal intensities from T2-weighted images of **(A)** normal liver parenchyma and **(B)** tumor before and after the injection of different agents. The error bars represent the standard deviation.

We then investigated the use of PLGA-coated Fe_3_O_4_ microcapsules as contrast agents in MR-guided HIFU ablation. As shown in the T_2_-weighted images in Figure 5 A_1,2_, B_1,2_ and C_1,2_, PLGA-coated Fe_3_O_4_ microcapsules generated negative contrast enhancement in the liver (Figure 5C_2_). Changes in the MR SI of the liver further demonstrated this effect. As shown in Figure 6, the SIs of liver parenchyma in the control, PLGA-coated Fe_3_O_4_ and PLGA groups before injection, and at 2 and 5 min after injection were as follows: 928.25 ± 17.41, 908.24 ± 17.38 and 921.04 ± 15.88 (pre-injection); 919.54 ± 18.28, 137.65 ± 15.23 and 938.39 ± 16.45 (2 min); and 925.59 ± 16.27, 271.34 ± 15.97 and 917.58 ± 16.47 (5 min), respectively. In addition, the SIs of liver tumors in the control, PLGA-coated Fe_3_O_4_ and PLGA groups before injection and at 2 and 5 min after injection were as follows: 921.37 ± 17.91, 918.76 ± 16.88 and 913.64 ± 15.88 (pre-injection); 924.65 ± 18.28, 783.64 ± 12.53 and 906.25 ± 16.45 (2 min); and 922.44 ± 18.37, 761.53 ± 13.57 and 908.52 ± 14.57 (5 min), respectively. The differences in the results were analyzed statistically between the groups and between the time points. Although there were no significant changes in SI after the injection of pure PLGA or no agent in either the normal liver parenchyma or the liver tumor at each time point (*P* > 0.05), the SI was significantly decreased in the normal liver parenchyma after administration of the PLGA-coated Fe_3_O_4_ microcapsules at 2 and 5 min relative to the other two groups (*P* < 0.05); however, the SI was decreased to a significantly lesser degree in the liver tumor tissue at 2 and 5 min relative to the other two groups (*P* < 0.05). These results demonstrated a significant SI-based imaging contrast between the normal liver parenchyma and tumor tissue with the use of PLGA-coated Fe_3_O_4_ microcapsules; this should enhance the imaging capabilities of MR during MR-guided HIFU. We hypothesize that this differential decrease in SI between the normal liver parenchyma and tumor tissue may be due to differential phagocytosis of the microcapsules by liver Kupffer cells; this is because PLGA-coated Fe_3_O_4_ microcapsules have the ability to distort local magnetic characteristics to yield a negative enhancement image. This SI-based effect is beneficial for MR-guided HIFU cancer surgery, because higher-contrast MR images facilitate the accurate localization of ultrasonic energy at the desired tumor site, resulting in improved therapeutic efficiency while substantially limiting damage to the surrounding normal tissue. Therefore, administration of the PLGA-coated Fe_3_O_4_ microcapsules could be used to localize the tumor using the variation in MR contrast between different tissues.

### PLGA-coated Fe_3_O_4_ microcapsules show synergy with MR-guided HIFU surgery

We also investigated the properties of PLGA-coated Fe_3_O_4_ microcapsules as synergistic agents for MR-guided HIFU liver cancer surgery. Dibaji et al. assessed the utility of using magnetic nanoparticles to enhance heating during HIFU procedures *in vitro*; their findings demonstrated that the introduction of magnetic nanoparticles could locally increase the temperature [36]. In the present study, PLGA-coated Fe_3_O_4_ microcapsules were intravenously administrated via the ear vein into rabbits with VX2 liver tumors. The microcapsules penetrated the liver tumor tissue as a result of the EPR effect and enhanced MR-guided HIFU ablation.

**Figure 7:**
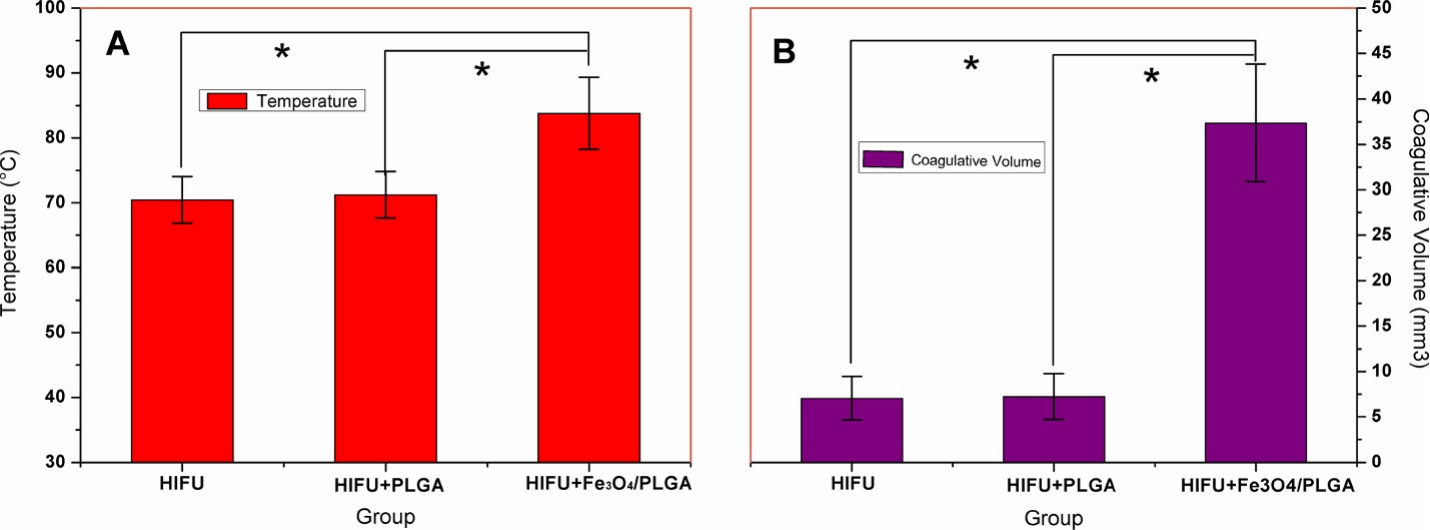
**Tissue temperature and coagulative volume analysis. (A)** Tissue temperatures of the different groups after MR-guided HIFU ablation. **(B)** Volume of coagulative necrosis in the different groups exposed to MR-guided HIFU. *P < 0.05.

**Figure 8:**
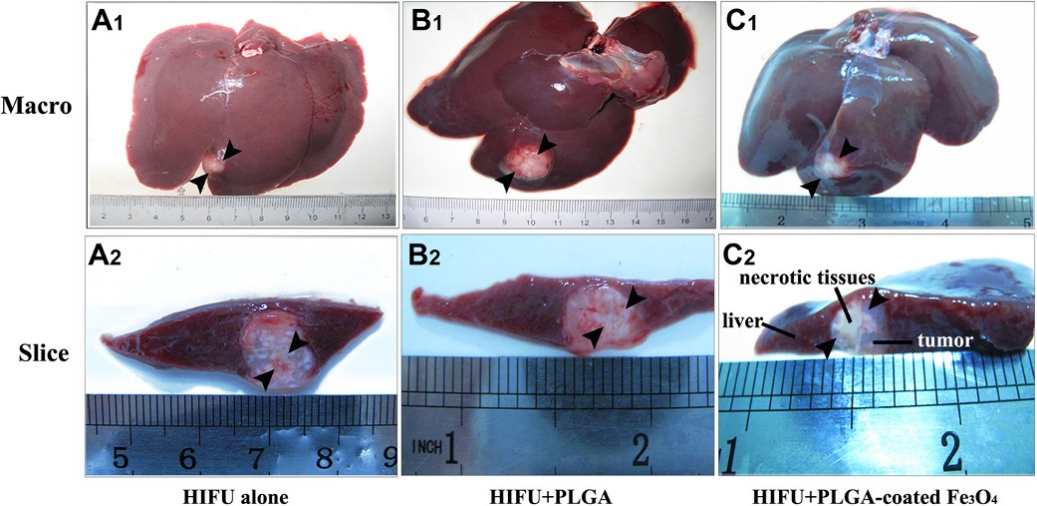
**Macroscopic inspection of liver tumors after MR-guided HIFU. (A**_**1**_ **/A**_**2**_ **)** MR-guided HIFU alone. **(B**_**1**_ **/B**_**2**_ **)** MR-guided HIFU + pure PLGA microcapsules. **(C**_**1**_ **/C**_**2**_ **)** MR-guided HIFU + PLGA-coated Fe_3_O_4_ microcapsules. Black arrows indicate the necrotic tissue in the liver tumors.

**Figure 9:**
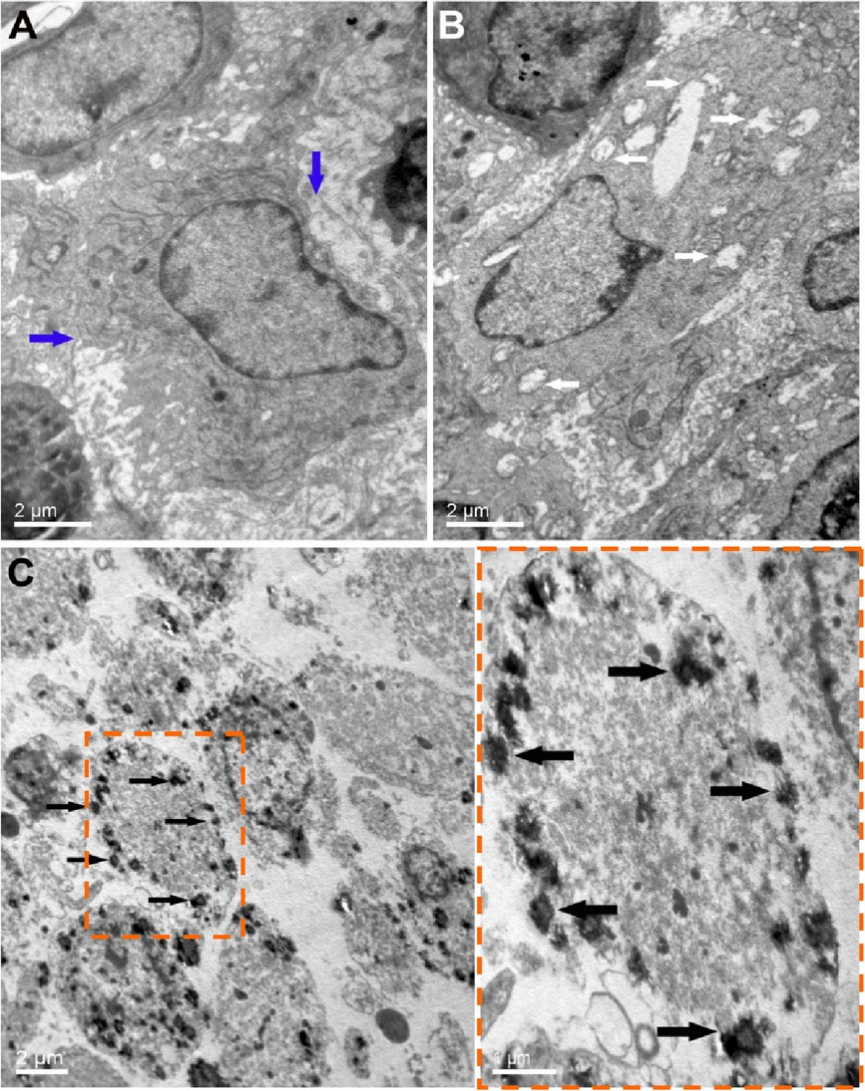
**Transmission electron microscope images of ablated tumor tissue. (A)** MR-guided HIFU alone. The blue arrows indicate the interrupted cell membranes. **(B)** MR-guided HIFU + pure PLGA microcapsules. The white arrows indicate the distended mitochondria. **(C)** MR-guided HIFU + PLGA-coated Fe_3_O_4_ microcapsules. The black arrows indicate the superparamagnetic PLGA-coated Fe_3_O_4_ microcapsules.

During MR-guided HIFU ablation, T-Map provided precise, real-time evaluation of the therapeutic effect. As shown in Figure 5 A_3_, B_3_ and C_3_, T-Map provided data on the extent of thermal ablation in the targeted tissue during treatment. Reliable thermal feedback from T-Map showed that the average temperature of the targeted tissue was raised by >70°C, indicating that the ultrasonic probe successfully focused a high-energy beam on the tumor tissue, resulting in the induction of coagulative necrosis. In addition, the average temperature of the ablated region was significantly higher in the MR-guided HIFU group using the PLGA-coated Fe_3_O_4_ microcapsules compared with the MR-guided HIFU group using pure PLGA microcapsules, or the MR-guided HIFU group without microcapsules (Figure 7A; *P* < 0.05). Moreover, the volume of coagulative necrosis was substantially larger in the MR-guided HIFU group with PLGA-coated Fe_3_O_4_ microcapsules relative to either the MR-guided HIFU group with pure PLGA microcapsules or the MR-guided HIFU group without microcapsules (*P* < 0.05; Figures 7B and 8). Six TEM images were compared in each group for investigation of the ultramicrostructure of the targeted tumor tissue after MR-guided HIFU ablation. The results (Figure 9) regarding tumor tissue indicated that: (i) MR-guided HIFU ablation was evident; (ii) ultrastructural changes were apparent; (iii) mitochondria were severely distended; and (iv) cell membranes and nuclear membranes were interrupted and undefined. In the MR-guided HIFU group with PLGA-coated Fe_3_O_4_ microcapsules, the microcapsules were deposited in the tumor cells, further demonstrating that they had entered the targeted tumor tissue.

## Conclusions

PLGA-coated Fe_3_O_4_ microcapsules combine the merits of the unique magnetic properties of SPIONs and the excellent biocompatibility of PLGA particles that make them attractive biomaterial candidates for MR-guided HIFU cancer ablation. These paramagnetic microcapsules exhibited contrast-enhanced imaging capability regarding MR imaging after intravenous injection; they effectively improved the accuracy of MR-guided HIFU liver cancer surgery. In addition, the introduction of PLGA-coated Fe_3_O_4_ microcapsules enhanced ultrasonic wave absorption and energy deposition in the targeted tissue, boosting hyperthermia in the targeted tissue and improving thermal ablation using MR-guided HIFU in the focused region. Furthermore, the PLGA-coated Fe_3_O_4_ microcapsules exhibited an excellent acute biosafety profile both *in vitro* and *in vivo*. However, the size of the superparamagnetic microcapsules used in the current study was slightly larger than those previously used. This was a limitation of our study and should be addressed in future investigations. In short, the administration of PLGA-coated Fe_3_O_4_ microcapsules provides an alternative strategy for MR-guided non-invasive HIFU synergistic therapy of cancer.

## Electronic supplementary material

Additional file 1: **Size distribution of the** **PLGA-coated** **Fe**_**3**_ **O**_**4**_ **microcapsules.** (TIFF 267 KB)
