# Supplementary figures and images for: Evaluation of superparamagnetic iron oxide-polymer composite microcapsules for magnetic resonance-guided high-intensity focused ultrasound cancer surgery
Source: BMC Cancer. 2014 Nov 3;14:800. doi: 10.1186/1471-2407-14-800 (PMC4228079; doi:10.1186/1471-2407-14-800)

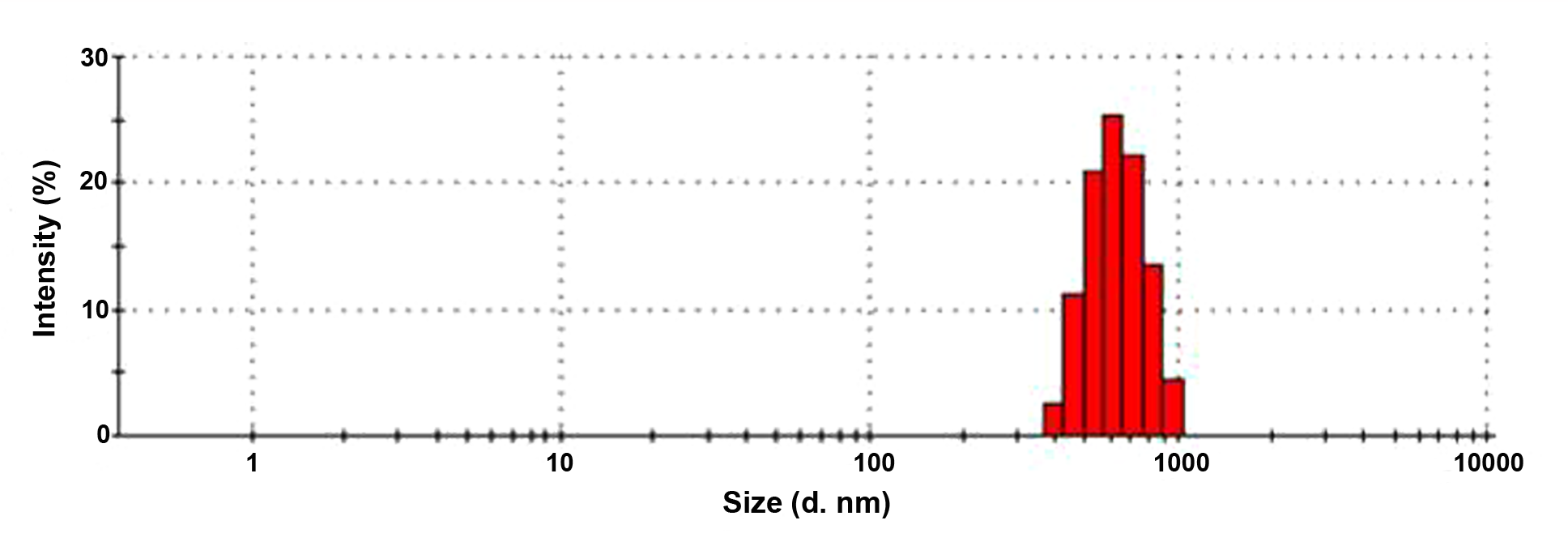

Supplement: Supplementary file 1 — Additional file 1: Size distribution of the PLGA-coated Fe 3 O 4 microcapsules. (TIFF 267 KB) [file 12885_2014_4974_MOESM1_ESM.tiff]
